# Ancient Grains as Functional Foods: Integrating Traditional Knowledge with Contemporary Nutritional Science

**DOI:** 10.3390/foods14142529

**Published:** 2025-07-18

**Authors:** Jude Juventus Aweya, Drupat Sharma, Ravneet Kaur Bajwa, Bliss Earnest, Hajer Krache, Mohammed H. Moghadasian

**Affiliations:** 1 Department of Food and Human Nutritional Sciences, University of Manitoba, Winnipeg, MB R3T 2N2, Canada; shar205@myumanitoba.ca (D.S.); bajwark1@myumanitoba.ca (R.K.B.); 2 St. Boniface Hospital Albrechtsen Research Centre, Winnipeg, MB R2H 2A6, Canadakracheh@myumanitoba.ca (H.K.); 3 Interdisciplinary Health Program, Rady Faculty of Health Sciences, University of Manitoba, Winnipeg, MB R3T 2N2, Canada

**Keywords:** ancient grains, functional foods, bioactive compounds, chronic disease prevention, dietary fiber, nutraceuticals, sustainable food systems, underutilized cereals

## Abstract

Ancient grains, including wild rice, millet, fonio, teff, quinoa, amaranth, and sorghum, are re-emerging as vital components of modern diets due to their dense nutritional profiles and diverse health-promoting bioactive compounds. Rich in high-quality proteins, dietary fiber, essential micronutrients, and a broad spectrum of bioactive compounds such as phenolic acids, flavonoids, carotenoids, phytosterols, and betalains, these grains exhibit antioxidant, anti-inflammatory, antidiabetic, cardioprotective, and immunomodulatory properties. Their health-promoting effects are underpinned by multiple interconnected mechanisms, including the reduction in oxidative stress, modulation of inflammatory pathways, regulation of glucose and lipid metabolism, support for mitochondrial function, and enhancement of gut microbiota composition. This review provides a comprehensive synthesis of the essential nutrients, phytochemicals, and functional properties of ancient grains, with particular emphasis on the nutritional and molecular mechanisms through which they contribute to the prevention and management of chronic diseases such as cardiovascular disease, type 2 diabetes, obesity, and metabolic syndrome. Additionally, it highlights the growing application of ancient grains in functional foods and nutrition-sensitive dietary strategies, alongside the technological, agronomic, and consumer-related challenges limiting their broader adoption. Future research priorities include well-designed human clinical trials, standardization of compositional data, innovations in processing for nutrient retention, and sustainable cultivation to fully harness the health, environmental, and cultural benefits of ancient grains within global food systems.

## 1. Introduction

Ancient grains refer to a diverse group of cereal and pseudocereal crops that have been cultivated for thousands of years and remained largely unchanged by modern agricultural practices. These grains include early domesticated cereals such as einkorn (*Triticum monococcum*), emmer (*Triticum dicoccum*), spelt (*Triticum spelta*), and Khorasan wheat (commercially known as KAMUT^®^), as well as regionally significant grains, such as millet, sorghum, barley, and teff, which continue to play vital roles in African and Asian diets [1]. Pseudocereals such as quinoa, amaranth, and buckwheat, though not true cereals taxonomically, are often grouped with ancient grains due to their similar nutritional value and historical significance [2]. These crops are increasingly recognized for their health benefits, environmental resilience, and contributions to dietary diversity.

Unlike modern grains, which have been selectively bred for high yield, uniformity, and processing efficiency, ancient grains have retained greater genetic diversity and structural integrity. They are typically consumed in whole or minimally processed forms, preserving the bran, germ, and endosperm. This results in superior nutritional profiles, including higher levels of dietary fiber, essential vitamins and minerals, and a wide array of bioactive phytochemicals such as polyphenols, flavonoids, and phytosterols [3]. These compounds are associated with improved metabolic health, antioxidant protection, and anti-inflammatory effects [4,5,6].

The use of many ancient grains, however, has declined over time due to the global shift toward industrialized agriculture, monocultures, and ultra-processed foods. This transition has contributed to the erosion of dietary diversity, rising micronutrient deficiencies, and increased prevalence of noncommunicable diseases. Reintegrating ancient grains into food systems offers an opportunity to improve both nutritional adequacy and public health, particularly in low-income and food-insecure regions [5,7].

Beyond their nutritional advantages, ancient grains also offer significant agronomic and environmental benefits. Their inherent genetic diversity allows adaptation to a wide range of abiotic and biotic stresses, including drought, poor soil fertility, and pests, traits often diminished in high-yield modern cultivars [2,5]. For example, millets thrive in arid climates, while wild rice regenerates naturally in aquatic ecosystems. Incorporating these crops into diversified farming systems enhances soil health, reduces reliance on agrochemicals, and supports ecological sustainability [8,9]

Culturally, ancient grains have sustained civilizations for millennia, underpinning traditional foodways, social practices, and food sovereignty. The cultivation and consumption of regionally adapted grains empower local communities, promote economic resilience, and preserve Indigenous and traditional knowledge [10,11]. Reviving these grains aligns with growing global movements to decolonize food systems, protect biodiversity, and promote culturally appropriate diets.

Given this intersection of genetic resilience, nutritional richness, environmental sustainability, and cultural significance, ancient grains are increasingly regarded as valuable assets for developing functional foods and advancing sustainable agriculture. Despite rising interest, comprehensive evaluations of their full nutraceutical potential, agronomic benefits, and sociocultural importance remain limited.

This review aims to provide an integrative assessment of ancient grain cereals and pseudocereals, examining their macronutrient and micronutrient composition, health-promoting bioactive compounds, and documented or potential roles in disease prevention and health promotion. We also explore their traditional uses, current applications in functional food development, and emerging market trends, while identifying challenges and opportunities for embedding ancient grains within sustainable, health-supportive food systems (Table 1).

## 2. Historical, Cultural, and Indigenous Significance of Ancient Grains

**Figure 1:**
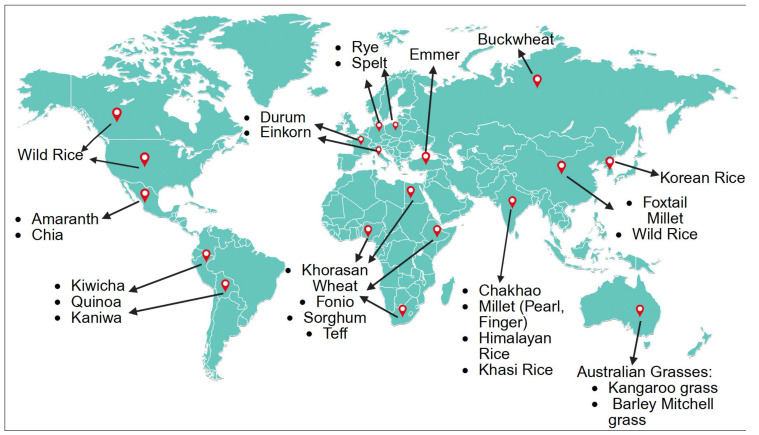
Global origins and distribution of ancient grains with functional food potential. This map illustrates the geographical origins and historical dissemination of key ancient grains, including quinoa, amaranth, millet, sorghum, teff, fonio, spelt, emmer, einkorn, wild rice, etc.

Ancient grains have nourished human societies for millennia, with their cultivation and use deeply embedded in the agricultural, cultural, and spiritual practices of diverse communities across the world (Figure 1). Despite the global shift toward industrial agriculture and modern staple crops, these grains continue to play vital roles in traditional food systems, Indigenous resilience, and emerging efforts to promote sustainable, plant-based diets. This section highlights the historical, cultural, and Indigenous significance of ancient grains across different regions.

### 2.1. Historical Significance: Foundations of Early Agriculture

The domestication of ancient grains formed the cornerstone of early agricultural societies in Africa, Asia, the Americas, Europe, and Australia. In Africa, teff (*Eragrostis tef)* has been cultivated in Ethiopia for over 3000 years, serving as a key component of food systems and agricultural sustainability [35,36]. Similarly, sorghum, millet, and barley were early staples across East Africa, contributing to the resilience of farming communities in arid environments [4,37,38]. In South Asia, small millets such as finger millet (*Eleusine coracana*), pearl millet (*Pennisetum glaucum*), and foxtail millet (*Setaria italica*) have long histories as drought-resilient staples essential for food security [28,39]. In northeastern India, traditional rice landraces such as Kba-khawlieh (Oryza sativa) reflect ancient agricultural diversity [21].

In the Americas, ancient grains such as quinoa (*Chenopodium quinoa*), amaranth (*Amaranthus* spp.), and chia (*Salvia hispanica* L.) were domesticated by Indigenous civilizations including the Inca, Aztec, and Maya [40,41,42,43,44]. These crops adapted to challenging environments, providing nutrition and fostering agricultural diversity. In Europe, grains such as einkorn (*Triticum monococcum*), emmer (*Triticum dicoccum*), spelt (*Triticum spelta*), barley, millet, and Khorasan wheat shaped prehistoric agricultural systems, as evidenced by archaeobotanical findings [45,46]. In Australia, Aboriginal peoples developed complex harvesting of native grasses long before the introduction of wheat, reflecting ancient relationships with the land [19,47].

### 2.2. Cultural Significance: Rituals, Traditions, and Identity

Ancient grains have also held deep cultural and symbolic importance in societies throughout history. In Ethiopia, teff is not only a dietary staple but central to cultural identity through the preparation of injera, a fermented flatbread central to Ethiopian cuisine and social life [35,36]. In South Asia and Africa, millet- and sorghum-based porridges, breads, and beverages are integral to culinary traditions, often linked to festivals and community gatherings [4,37,38].

In Central and South America, quinoa and amaranth were traditionally revered in religious ceremonies and rituals, embodying the spiritual connection between people and the land [40,41,42]. Chia was similarly used in spiritual offerings and as sustenance for warriors, underscoring its cultural value among the Aztec and Maya [43,44]. In North America, wild rice (*Zizania* spp.) holds profound ceremonial importance for Indigenous nations, notably the Anishinaabe, where it features in spiritual practices as well as sustenance [48,49,50].

In Europe, ancient grains were central to early societies not only for nutrition but also in traditional baking and brewing, with historical breads and fermented products reflecting regional identities [45,46]. In Australia, native grains were woven into cultural practices, ceremonies, and community knowledge systems long before colonization, demonstrating the inseparability of food and culture [19,47].

### 2.3. Indigenous Significance: Knowledge, Sovereignty, and Sustainability

For Indigenous communities, ancient grains are not only nutritional resources but living embodiments of ecological knowledge, resilience, and sovereignty. In Africa and Asia, the preservation of traditional grains such as sorghum, millets, and heritage rice varieties support biodiversity, climate resilience, and food security [28,39]. Traditional fermentation techniques enhance nutritional benefits and reflect Indigenous food wisdom [51,52].

In the Americas, renewed interest in ancient grains such as quinoa, amaranth, chia, and wild rice aligns with movements to reclaim Indigenous food sovereignty, restore ancestral foodways, and promote environmental stewardship [40,41,42,43,44,48,49,50,53,54,55,56]. Chia, now globally recognized as a “superfood,” highlights the scientific validation of Indigenous knowledge that long appreciated its medicinal and nutritional value [53,54,55,56].

In Europe, the revival of ancient grains supports sustainable farming, biodiversity, and traditional food cultures [4,57,58]. In Australia, Indigenous-led initiatives to reintroduce native grains like kangaroo grass and Mitchell grass are advancing not only nutrition and ecological restoration but also the cultural integrity and autonomy of Aboriginal communities [19,47,59].

**Figure 2:**
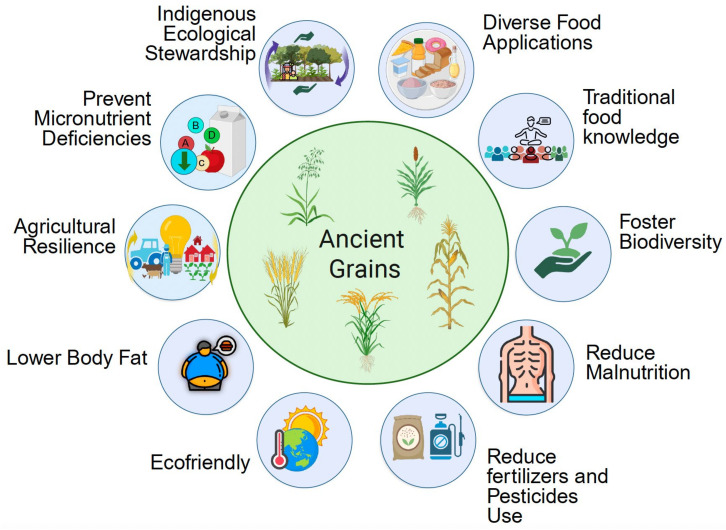
Ancient grains and their interconnected nutritional, cultural, and agronomic significance.

Across different regions, ancient grains have shaped human history, culture, and resilience, serving not only as vital sources of sustenance but also as powerful symbols of identity, spirituality, and ecological knowledge. Their continued cultivation and revival reflect the enduring importance of traditional food systems, Indigenous knowledge, and biodiversity in addressing contemporary challenges related to food security, nutrition, and sustainability (Figure 2). By recognizing and valuing the historical, cultural, and Indigenous significance of these grains, there is an opportunity to build more resilient, equitable, and health-promoting food futures.

## 3. Health-Promoting Potential of Ancient Grains

Ancient grains are increasingly valued not only for their cultural and agronomic significance but also for their rich nutritional profiles and wide-ranging health benefits. Unlike many modern cereals, ancient grains are typically cultivated using traditional agroecological methods and are most often consumed in whole or minimally processed forms. This practice helps preserve their inherent nutrient density and diverse phytochemical composition, which together contribute to their growing reputation as functional foods with both preventive and therapeutic potential.

Mounting evidence highlights the multifaceted health-promoting properties of ancient grains, including their contributions to cardiovascular health, glycemic regulation, immune function, and the prevention and management of chronic diseases such as diabetes, obesity, metabolic syndrome, and micronutrient deficiencies. These effects are largely attributable to their superior content of high-quality proteins, dietary fiber, essential fatty acids, minerals, vitamins, and bioactive phytochemicals with antioxidant and anti-inflammatory properties.

To explore these benefits, this section first examines the nutritional composition, functional properties, and health mechanisms of ancient grains. This is then followed by exploring their roles in specialized diets, disease management, and broader health promotion, with an emphasis on their applications in addressing specific dietary needs and public health challenges.

### 3.1. Nutritional Composition, Functional Properties, and Health Mechanisms of Ancient Grains

Ancient grains are distinguished by exceptional nutritional density and diverse phytochemical composition, setting them apart from many modern staple crops. Consumed primarily in whole or minimally processed forms, these grains provide a rich array of essential macronutrients, such as high-quality proteins, dietary fiber, and beneficial fatty acids, alongside vital micronutrients such as iron, magnesium, calcium, zinc, and B vitamins. They are also rich in bioactive compounds including phenolic acids, flavonoids, anthocyanins, carotenoids, saponins, and phytosterols, which together support antioxidant, anti-inflammatory, and metabolic regulatory effects [4,60,61]. This subsection offers an integrated overview of these essential nutrients and bioactive compounds, alongside analysis of their functional health benefits across different ancient grain species.

#### 3.1.1. Macronutrient Composition and Protein Quality

Many ancient grains contain higher protein levels than modern wheat and rice, with some also providing complete amino acid profiles. For instance, ancient wheat species such as einkorn (*Triticum monococcum*), emmer (*Triticum dicoccum*), spelt (*Triticum spelta*), and Khorasan wheat (KAMUT^®^) offer 11–17% protein alongside beneficial minerals [62,63]. Similarly, Andean pseudocereals including quinoa (*Chenopodium quinoa*), amaranth (*Amaranthus* spp.), and kañiwa (*Chenopodium pallidicaule*) are especially notable for their complete amino acid profiles, providing all essential amino acids including lysine, often limited in cereal grains [64]. Chia (*Salvia hispanica*) and tarwi (*Lupinus mutabilis*) are valued for their rich protein and beneficial fat content, making them beneficial for plant-based and specialized diets [44,53,64].

#### 3.1.2. Dietary Fiber, Glycemic Control, and Gut Health

Ancient grains are valuable sources of dietary fiber, including soluble fibers such as β-glucans (in barley and oats) and resistant starch and arabinoxylans (in millets, pigmented rice, teff, and wild rice), which are instrumental in supporting glycemic control, gut microbiota modulation, and digestive health [4,14,65]. Regular consumption of these fibers has been associated with improvement in metabolic disorders, including insulin sensitivity, cholesterol reduction, and reduced risk of type 2 diabetes [66].

#### 3.1.3. Micronutrient Density and Mineral Bioavailability

Compared to modern cereals, ancient grains are superior sources of essential minerals, including iron, magnesium, calcium, zinc, and selenium, vital for anemia prevention, bone health, immune function, and enzymatic processes [60,61]. Einkorn and Khorasan wheat are particularly high in iron and magnesium [33], while Teff (*Eragrostis tef*), millets, and wild rice are notable for their high iron and calcium content. Similarly, Australian native grains such as Mitchell grass and kangaroo grass are exceptional for their zinc and iron levels [19,48,67,68]. These grains offer dietary solutions to combat micronutrient deficiencies, especially in vulnerable populations.

#### 3.1.4. Beneficial Fatty Acids and Cardiometabolic Health

Several ancient grains provide beneficial unsaturated fatty acids that support cardiovascular and metabolic health. Chia seeds are exceptionally rich in alpha-linolenic acid (ALA), a plant-based omega-3 fatty acid with potential anti-inflammatory and cardioprotective effects [69,70]. Similarly, amaranth, quinoa, and tarwi provide health-promoting fatty acids, which support lipid profile improvement and enhance cardiovascular and metabolic health [64].

#### 3.1.5. Phytochemical Richness and Antioxidant Capacity

A distinguishing feature of ancient grains is their rich phytochemical composition, including phenolic acids, flavonoids, anthocyanins, saponins, carotenoids, phytosterols, and avenanthramides [13,48]. These bioactives contribute to potent antioxidant, anti-inflammatory, antidiabetic, and potential anticancer effects. For example, pigmented rice varieties (black, red, and brown) are especially rich in anthocyanins and phenolic acids, which enhance their antioxidant capacity and modulate metabolic pathways [24,26]. Similarly, the pigmented rice varieties *Mappillai Samba* and *Kataribhog* are rich in anthocyanins and resistant starch, enhancing their glycemic and oxidative stress-modulating effects [12,14], whereas sorghum contains 3-deoxyanthocyanidins, unique polyphenolic compounds with anti-inflammatory activity and potential anticancer effects [71]. Ancient wheats such as spelt, emmer, and einkorn contain higher polyphenol levels than modern wheat, which are linked to reduced oxidative stress and inflammatory cytokine production [32,59]. Similarly, chia seeds offer a wide spectrum of polyphenols, including quercetin and chlorogenic acid, which work synergistically with fiber and unsaturated fats to lower oxidative stress and improve glycemic response [44,54]. Traditional rice landraces (e.g., *Karunguruvai*, *Kichili Samba*) are rich in tocopherols, terpenoids, phytosterols, and γ-oryzanol, linked to cholesterol reduction, immune support, and anti-inflammatory effects [72,73].

#### 3.1.6. Nutritional Mechanisms of Ancient Grains

The health-promoting effects of ancient grains are mediated through multiple, interconnected biological pathways driven by their dense content of dietary fiber, polyphenols, flavonoids, phenolic acids, anthocyanins, carotenoids, phytosterols, bioactive peptides, essential fatty acids, and micronutrients. These compounds exert antioxidant effects by scavenging reactive oxygen and nitrogen species and upregulating endogenous antioxidant enzymes, thereby reducing oxidative stress and cellular damage [38,59,74]. The anti-inflammatory actions of ancient grains are achieved through the inhibition of NF-κB signaling and suppression of pro-inflammatory cytokines (TNF-α, IL-6), alongside immune modulation [26,75].

Dietary fibers such as β-glucans and resistant starches modulate glycemic response, improve lipid metabolism, and activate key pathways including AMP-activated protein kinase (AMPK) and peroxisome proliferator-activated receptors (PPARs) [13,26], contributing to metabolic regulation and cardiovascular health [76,77]. Ancient grains also shape gut microbiota composition, enriching beneficial microbes and promoting the production of short-chain fatty acids (SCFAs), which enhance gut barrier integrity and systemic metabolic outcomes [78,79,80].

Emerging evidence suggests potential roles of ancient grains in mitochondrial biogenesis and cellular energy metabolism, further supporting metabolic homeostasis [77,81]. The cumulative and synergistic action of these bioactive components underpins the observed protective effects of ancient grains against chronic diseases such as type 2 diabetes, cardiovascular disease, obesity, and certain cancers [6,59,82].

Collectively, the superior nutrient density, bioactive composition, and multifunctional health properties of ancient grains highlight their potential as foundational ingredients in the development of functional foods and health-oriented diets. Building on these essential attributes, the following subsection explores how ancient grains can be strategically integrated into specialized diets and public health interventions to address specific nutritional needs and manage chronic diseases.

### 3.2. Role in Specialized Diets, Disease Management, and Health Promotion

The superior nutrient and phytochemical composition of ancient grains supports their growing use in specialized diets and health promotion strategies aimed at addressing both undernutrition and the global rise in non-communicable diseases. Their compatibility with diverse dietary patterns, such as gluten-free, plant-based, low-glycemic, and culturally specific diets, makes them valuable components in managing conditions such as diabetes, cardiovascular disease, obesity, and micronutrient deficiencies. This subsection examines the applications of ancient grains in various dietary interventions and public health contexts, highlighting their role in chronic disease prevention, therapeutic nutrition, and the advancement of sustainable, culturally inclusive food systems.

#### 3.2.1. Specialized Diets and Nutrient-Dense Alternatives

Ancient grains could be highly compatible with specialized diets, including plant-based, gluten-free, low-glycemic, and allergen-sensitive regimens. Many varieties such as quinoa, amaranth, chia, teff, millets, and wild rice are naturally gluten-free and rich in essential amino acids, making them potentially suitable for individuals with celiac disease, gluten sensitivity, or vegetarian and vegan dietary preferences [64,83].

Chia seeds (*Salvia hispanica* L.), notably, provide high-quality plant protein, omega-3 fatty acids, and bioactive polyphenols, supporting cardioprotective, antidiabetic, and anti-inflammatory effects [44,69]. Their gelling and emulsifying properties further enhance their functional applications in diverse food products such as baked goods, beverages, and baby foods [84]. Similarly, Andean grains such as quinoa, kañiwa, and tarwi offer complete protein profiles, high micronutrient density, and are well-suited for muscle maintenance, immune health, and metabolic balance in plant-based diets [85].

In populations at risk of micronutrient deficiencies, ancient grains such as fonio, teff, and millets serve as valuable sources of iron, zinc, calcium, and B vitamins, helping to combat “hidden hunger” and contributing to food security in low-income and food-insecure settings [27]. Furthermore, some micronutrients, such as vitamin B12 and vitamin E, often lacking in plant-based or allergen-sensitive diets, can be partially complemented through the inclusion of ancient grains that supply such critical micronutrients, thereby reducing the risk of nutrient insufficiency [86,87].

#### 3.2.2. Chronic Disease Management and Functional Properties

Emerging evidence highlights the role of ancient grains in managing chronic diseases such as diabetes, cardiovascular disorders, obesity, and gastrointestinal conditions. The low glycemic index and high dietary fiber content of many ancient grains, including Australian native grains and pigmented rice varieties, have demonstrated efficacy in improving glycemic control, insulin sensitivity, and lipid metabolism [13,20,88]. Wild rice consumption, for example, has been associated with reduced plasma cholesterol levels, attenuation of atherosclerosis, and improved metabolic markers in animal studies [15,16,89].

Pigmented rice varieties, such as red and black rice, contain anthocyanins and other phenolic compounds that modulate adipogenesis, enhance antioxidant defenses, and support metabolic health through activation of key pathways including AMPK and PPARs [26,88]. Similarly, bioactives in sorghum, chia, and barley have been shown to confer anti-inflammatory, hypolipidemic, and chemopreventive effects [54,71,90].

Importantly, many phytochemicals present in ancient grains are not fully absorbed in the upper gastrointestinal tract but are metabolized by gut microbiota in the colon, generating bioactive metabolites that exert systemic antioxidant, anti-inflammatory, and gut-protective effects [91,92]. This prebiotic potential adds to the growing recognition of ancient grains in promoting gut health and supporting the gut–immune axis.

#### 3.2.3. Integration into Functional Foods and Sustainable Diets

Ancient grains are increasingly incorporated into functional foods such as whole-grain breads, cereals, and snack products, providing opportunities to enhance nutritional value while catering to health-conscious consumers [93]. Baked goods made from ancient wheats such as einkorn, Khorasan, and spelt demonstrate higher antioxidant capacity compared to those made with modern wheat, particularly when fortified with herbs or other bioactive-rich ingredients [93].

Their environmental resilience, low input requirements, and cultural significance also make ancient grains key contributors to sustainable food systems and Indigenous food sovereignty [19,94]. Incorporating culturally appropriate grains into public health and nutrition-sensitive interventions can improve dietary equity and community well-being, particularly in Indigenous and rural populations (Figure 2).

The integration of ancient grains into specialized diets not only supports the management of chronic conditions but also contributes to dietary sustainability, cultural preservation, and nutrition equity. Together, these benefits underscore the relevance of ancient grains in advancing global health initiatives and building resilient, health-promoting food systems.

## 4. Functional Food Applications of Ancient Grains

The growing consumer demand for plant-based, clean-label, and health-enhancing food products has catalyzed innovation in functional food development [95,96]. Ancient grains meet this demand as some offer bioactive-rich alternatives that are naturally gluten-free, such as amaranth, quinoa, millet, sorghum, teff, and buckwheat, have a low glycemic index, rich in slow-digesting carbohydrates, essential amino acids, dietary fiber, and minerals [42,97,98]. Furthermore, many ancient grains possess enzyme-inhibitory activities (e.g., α-amylase and α-glucosidase inhibition), which help regulate blood glucose levels and improve postprandial metabolic responses. These characteristics make ancient grains valuable ingredients in developing foods targeted toward individuals with metabolic disorders, dietary intolerances, and nutrient deficiencies.

To explore these aspects in greater detail, the following subsections examine (1) the current strategies for incorporating ancient grains into various food matrices, and (2) key processing innovations and challenges that influence the retention of nutritional and functional qualities during product development.

### 4.1. Applications, Innovations, and Processing Strategies

Ancient grains have found wide-ranging applications in contemporary food systems due to their superior nutritional quality, functional versatility, and compatibility with health-oriented product development. These grains are increasingly incorporated across multiple product categories, such as baked goods, beverages, fermented foods, and functional snacks, offering both technological and nutritional advantages.

**Bakery Products:** These products represent a major application area, where grains such as spelt, einkorn, emmer, barley, KAMUT^®^, and heirloom corn varieties are used to enrich breads, flatbreads, crackers, and cookies with dietary fiber, high-quality protein, and micronutrients. Processing techniques such as germination and sourdough fermentation are commonly employed to improve dough rheology, sensory properties, and the bioavailability of key nutrients by reducing antinutritional factors, such as phytic acid and tannins. Nonetheless, these grains’ inherently low gluten content and unique starch characteristics require careful formulation adjustments to maintain optimal texture, elasticity, and shelf-life [34,99].

**Functional beverages:** Beverages incorporating ancient grains, such as oat- and rice-based milk alternatives, quinoa-based drinks, and barley infusions, are gaining popularity due to their content of bioactive peptides, polyphenols, and dietary fiber. These components contribute to a range of health benefits, including antioxidant, antihypertensive, and digestive effects [100,101]. Similarly, fermented pseudocereals like quinoa and amaranth, especially those fermented with lactic acid bacteria, are enriched with probiotic strains. These foods offer lactose-free and gut-friendly options, enhancing digestibility and reducing antinutritional factors, making them appealing to consumers seeking plant-based alternatives [102,103,104].

**High-fiber, high-protein snacks:** These are snacks, usually innovated with ancient grains, and often in combination with upcycled ingredients such as brewers’ spent grain and cowpea flour, address consumer demand for nutrient-dense, sustainable, and minimally processed foods. They promote satiety, support metabolic health, and contribute to circular food economy by reducing food system waste [105].

Despite these promising applications, several challenges complicate the broader integration of ancient grains into mainstream functional food systems. Processing limitations arise from their distinctive kernel structures, protein matrices, and starch properties, which can affect hydration, dough formation, and textural consistency. Optimizing milling, drying, and fermentation methods is essential to ensure nutrient retention and desirable sensory attributes [60,99].

Moreover, product stability and shelf-life remain concerns, particularly in formulations enriched with fiber and polyphenols, which may be susceptible to flavor degradation, discoloration, and textural shifts over time. Stringent control of moisture, pH, and temperature during both processing and storage is necessary to maintain consumer appeal [102].

From an agronomic and economic standpoint, many ancient grains exhibit lower yields and are primarily grown in small-scale, traditional farming systems. These factors, along with limited mechanization and a lack of standardized processing protocols, contribute to supply chain inconsistencies, higher production costs, and relatively low market penetration [60,106]. Addressing these constraints is vital for scaling up their use in commercial product development and advancing the global functional food sector.

### 4.2. Market Adoption, Consumer Appeal, and Commercial Potential

Consumer interest in ancient grains is being driven by growing awareness of their health benefits, sustainable production practices, and cultural heritage. These factors have made ancient grains central to innovation in the functional food market, particularly within clean-label, organic, and plant-based product categories.

Ancient grains are widely perceived as wholesome, nutrient-dense, and gut-friendly, with attributes that support metabolic health, immune function, and chronic disease prevention. Their naturally high fiber content, low glycemic index, and some, such as amaranth, quinoa, millet, sorghum, and teff being gluten-free or having low-gluten profiles make them appealing to health-conscious consumers, as well as individuals with specific dietary needs, such as those managing celiac disease, gluten sensitivities, or vegetarian/vegan diets [101].

Beyond their health benefits, their sustainability and ethical appeal are increasingly recognized. Many ancient grains are cultivated using traditional, low-input agricultural practices that preserve biodiversity and reduce dependence on synthetic fertilizers and pesticides. These characteristics enhance their association with regenerative agriculture and environmentally responsible food systems, reinforcing their market attractiveness [60].

The market value of ancient grains is further amplified through product innovation and branding strategies. Ingredients such as chia, quinoa, teff, and millet are prominently featured in an array of functional foods, ranging from breakfast cereals and snack bars to non-dairy beverages, due to their content of omega-3 fatty acids, soluble fiber, antioxidants, and bioavailable minerals [107]. The “heritage grain” label resonates with consumers seeking authenticity, provenance, and cultural connection in their food choices.

Successful commercial products demonstrate the versatility and appeal of ancient grains in meeting modern dietary demands. For example, sprouted millet and quinoa cereals have been shown to improve nutrient bioavailability, while probiotic-rich amaranth yogurts support gut health through functional fermentation. Additionally, barley- and sorghum-based snacks, often formulated using upcycled grain materials, contribute to sustainability goals by reducing food waste and supporting circular economy principles [106,108,109,110,111].

Collectively, these trends reflect the strong alignment of ancient grains with consumer expectations around health, sustainability, and dietary inclusivity. By integrating ancient grains into innovative, science-backed food products, the industry can capitalize on shifting food system priorities while simultaneously addressing public health challenges and advancing global food and nutrition security.

## 5. Conclusions and Future Perspectives

Ancient grains, including wild rice, millet, fonio, teff, quinoa, and sorghum, represent a nutritionally rich and functionally diverse group of cereals and pseudocereals with deep cultural heritage and growing scientific, public health, and sustainability relevance. As detailed in this review, these grains are distinguished by their superior profiles of essential macronutrients (notably high-quality proteins, dietary fiber, and beneficial fatty acids), micronutrients (such as iron, magnesium, zinc, and B vitamins), and a diverse spectrum of bioactive phytochemicals, including phenolic acids, flavonoids, carotenoids, phytosterols, and betalains.

The health-promoting effects of these grains are underpinned by the synergistic actions of their nutrients and phytochemicals across multiple, interconnected biological pathways. These mechanisms include antioxidant defense, anti-inflammatory action, metabolic regulation of glucose and lipid homeostasis, mitochondrial function, and modulation of the gut microbiota. Collectively, these effects contribute to the prevention and management of chronic noncommunicable diseases such as cardiovascular disease, type 2 diabetes, obesity, and certain cancers. Furthermore, the naturally low glycemic index and gluten-free or low-gluten characteristics of several ancient grains make them especially suitable for individuals with metabolic disorders, celiac disease, or gluten sensitivities.

The strategic integration of ancient grains into modern dietary patterns, through public health guidelines, school feeding programs, and the development of functional foods, offers significant potential to enhance dietary diversity, improve micronutrient intake, and reduce the burden of diet-related chronic diseases. However, the broader adoption of ancient grains is challenged by technological limitations in processing and formulation, variability in sensory and nutritional qualities, limited agronomic scalability, and inconsistent consumer awareness or acceptance. Addressing these barriers will be essential for translating their nutritional potential into accessible, sustainable food solutions.

Future research should prioritize well-designed human clinical trials to substantiate the disease-preventive effects of ancient grains and clarify the specific molecular mechanisms by which their bioactive compounds exert health benefits. Most especially, studies exploring synergistic interactions among grain-derived phytochemicals, dietary fibers, and host metabolism, including gut microbiota modulation, are warranted. Additionally, efforts to standardize compositional data, optimize processing techniques to preserve or enhance bioactive content, and evaluate nutrient bioavailability across diverse populations will strengthen the evidence base.

At the food systems level, investments in the conservation of genetic diversity, agroecological cultivation, and sustainable value chains, particularly in regions rich in underutilized grain biodiversity such as sub-Saharan Africa, South Asia, and Latin America, will be critical to supporting resilient local economies, biodiversity preservation, and food security.

Ultimately, ancient grains exemplify the convergence of traditional food knowledge and contemporary nutritional science. As culturally significant, nutritionally superior, and ecologically resilient staples, they offer a promising and practical pathway toward healthier diets, inclusive food systems, and more sustainable agricultural futures.

## Figures and Tables

**Table 1:** Summary of Selected Ancient Grains and Their Nutritional-Bioactive Profiles and Health Benefits.

Grain Type	Notable Nutrients/Bioactives	Documented/Proposed Health Benefits	Selected References
Traditional Red and Pigmented Rice (e.g., *Mappillai Samba*, Chakhao, YZ6H)	Phenolic acids, flavonoids, tocopherols, phytosterols, squalene, anthocyanins, vitamins, minerals	Antioxidant, anti-inflammatory, anticancer, antihypercholesterolemic, neuroprotective	[12,13,14]
Wild Rice (*Zizania* spp.)	Protein, fiber, vitamins B/E, minerals (Fe, Zn, Mg), phenolics, phytosterols, γ-oryzanol	Anti-atherogenic, antidiabetic, hypocholesterolemic, metabolic and gut health benefits	[15,16,17,18]
Australian Native Grains	Protein, polyunsaturated fatty acids, phenolics, Ca, Fe, Zn, Mg	Glycemic control, cardiovascular and metabolic health, antioxidant, and anti-inflammatory	[19,20]
Indigenous and Aromatic Rice (India, Khasi, Himalaya)	Protein, resistant starch, fiber, iron, zinc, phenolics, aroma compounds	Low GI, antioxidant, digestive and metabolic benefits, ethnomedicinal value	[21,22,23]
Thai and Korean Pigmented Rice Bran	Anthocyanins, flavonoids, tocopherols, γ-oryzanol, fatty acids	Antioxidant, anti-obesity, antidiabetic, immune-modulatory	[24,25,26]
Teff, Fonio, Sorghum, Pearl Millet (African Grains)	Iron, zinc, calcium, vitamin A, B12, fiber, polyphenols	Combat malnutrition, manage NCDs, promote dietary diversity and food security	[27,28]
Kañiwa, Quinoa, Kiwicha (Andean Grains)	Protein, essential amino acids, fiber, phenolics, flavonoids, betalains	Antioxidant, anti-inflammatory, anticancer, colon health	[29,30]
Ancient Wheats (Einkorn, Emmer, Khorasan, Spelt)	Protein, fiber, polyphenols, minerals (Zn, Fe, Mg), MUFA, tocopherols	Antioxidant, anti-inflammatory, gut health, higher nutritional density than modern wheat	[31,32,33]
Oats, Buckwheat, Rye	β-glucans, resistant starch, phenolics, minerals	Glycemic control, cholesterol-lowering, antioxidant and cardiovascular health	[34]

Abbreviations: GI, glycemic index; NCDs, noncommunicable diseases; MUFA, Monounsaturated fatty acids.

## Data Availability

Data sharing is not applicable to this article as no new data were created or analyzed in this study.

## Abbreviations

The following abbreviations are used in this manuscript:

AMPK	AMP-activated protein kinase
FDA	U.S. Food and Drug Administration
GI	glycemic index
IL-6	interleukin-6
PPAR	Peroxisome proliferator-activated receptor
RNS	reactive nitrogen species
ROS	reactive oxygen species
TNF-α	tumor necrosis factor-alpha
Zn, Fe, Ca	Zinc, iron, calcium
